# Aziridination-Enabled Structural and Quantitative Lipidomics

**DOI:** 10.1021/jasms.6c00107

**Published:** 2026-07-25

**Authors:** Erin A. Hirtzel, Gopal Reddy Ramidi, Xin Yan

**Affiliations:** Department of Chemistry, 14736 Texas A&M University, College Station 77843, Texas, United States

**Keywords:** lipidomics, mass spectrometry, aziridination

## Abstract

Chemical derivatization has long been employed to enhance the structural characterization of lipids by mass spectrometry (MS). In recent years, olefin aziridination has emerged as a powerful and versatile strategy in lipidomics, driven by its ability to selectively target carbon–carbon double bonds (C=C bonds) and to introduce nitrogen-containing functionalities that facilitate both structural elucidation and quantitative analysis. Aziridination-enabled MS approaches provide reliable C=C bond localization through diagnostic fragmentation, while simultaneously improving ionization efficiency, particularly for nonpolar lipid classes. A diverse range of aziridination chemistry has been developed, each offering distinct advantages for lipid analysis. In this review, we summarize recent advances in aziridination-enabled MS methodologies, with an emphasis on reaction development and analytical performance. We further highlight applications across biological and complex sample systems. These developments highlight aziridination-assisted MS as a powerful strategy for precision lipidomics with isomer-resolved capability and accurate quantification.

## Graphical abstract

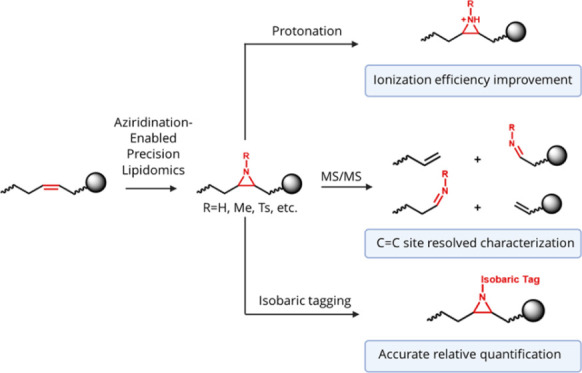

## Introduction

The study of lipids has gained increased interest in recent years, as lipids play critical roles in energy storage, cell signaling, and cell membrane structure.^1–4^ Perturbations in the lipidome have been linked to a variety of diseases and can have implications for disease detection,^5–10^ progression,^11–13^ and function.^14–17^ The wide range of lipids’ roles in biological systems is reflected in their diverse structures.^18,19^ Diseases can have major effects on lipid metabolism by changing the bioavailability of lipids and dysregulating synthetic pathways.^12,20–22^ Moreover, they can alter the quantities of specific lipid classes^23,24^ and influence the distribution of lipid isomers,^24,25^ underscoring the importance of both quantification and characterization in advancing lipidomics. Mass spectrometry (MS) has emerged as a powerful lipidomic tool, as its high specificity and sensitivity is advantageous in measuring spatial and temporal alterations in the lipidome.

Because the locations of carbon–carbon double bonds (C=C bonds) and the *sn*-positions of aliphatic chains on the glycerol backbone cannot be identified by conventional MS alone (Figure 1), many methods have emerged for the characterization of these lipid isomers. Gas-phase ion activation methods such as ozone-induced dissociation (OzID),^26–28^ ultraviolet photodissociation (UVPD),^29–32^ gas-phase charge inversion ion/ion reactions^33^ radical-directed dissociation (RDD)^34,35^ electron impact excitation of ions from organics (EIEIO),^36,37^ and oxygen attachment dissociation (OAD)^38–40^ can be used to elucidate C=C bond location by initiating alternative fragmentation patterns to provide structural information. Chemical derivatization methods such as olefin epoxidation,^41–46^ Paternò–Büchi (PB),^47–49^ cross-metathesis (CM),^50,51^ charge-switch *N*-(4-aminomethylphenyl) pyridinium (AMPP) derivatization,^52^ singlet oxygen (^1^ ΔO_2_)-ene reactions,^53,54^ light-catalyzed lipid double-bond cycloaddition,^55^ and click inverse electron demand Diels–Alder^56^ enable C=C bond localization by introducing functional groups that yield diagnostic fragments upon collision-induced dissociation (CID).

**Figure 1:**
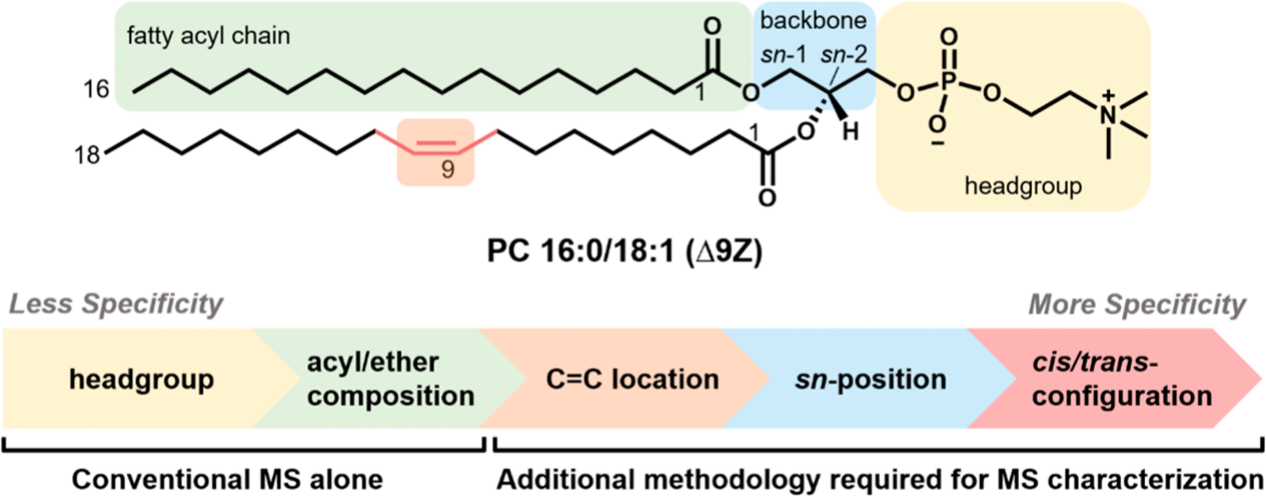
Representative lipid structure illustrated by PC 16:0/18:1 (9Z), highlighting different levels of structural identification and corresponding nomenclature, including the headgroup, acyl/ether chain composition, C=C bond location, *sn* position, and C=C configuration.

Distinguishing between *sn*-positional isomers traditionally relies on comparing the relative abundances of fatty acyl ions at the *sn*-1 and *sn*-2 positions, which are generated under CID.^4^ Methods for *sn*-isomer characterization include EIEIO,^57^ consecutive CID and OzID (CID/OzID),^28^ PB with MS^3^ (PB-MS^3^),^25^ RDD,^34,58,59^ and hybrid collisional activation/UVPD.^29^ Techniques leveraging ion mobility spectrometry (IMS), such as field asymmetric ion mobility spectrometry (FAIMS-MS, also known as differential mobility mass spectrometry, DMS-MS),^60–62^ liquid chromatography ion mobility mass spectrometry (LC-IM-MS),^63^ liquid chromatography-trapped ion mobility spectrometry-mass spectrometry (LC-TIMS-MS),^64^ have been demonstrated to provide *sn*-isomer resolution through separating lipid isomers in complex biological samples.

The traditional approach for accurate quantification of lipids relies on the use of stable isotope-labeled internal standards (ISs) to correct for analytical variability arising from matrix effects.^65^ Quantitative methods often incorporate chromatographic separation to improve accuracy and enhance the detection of low-abundance lipids; alternatively, direct infusion methods leveraging tandem (MS/MS) and high mass accuracy instrumentation have also been established.^65–67^ A key limitation of IS-based quantification is that accuracy depends on the availability of chemically identical standards for each analyte.^66^ Given both the challenge and importance of accurate quantification, the field of lipidomics has much to gain from the development of new quantitative approaches.

Another challenge in MS analysis of lipids is variability in ionization efficiency. While certain lipid classes, such as glycerophospholipids (GPLs), ionize readily by electrospray ionization and are detected with high sensitivity, others—such as triglycerides (TGs), sterols (STs), and cholesteryl esters (CEs)—ionize poorly and are more difficult to detect. Gas chromatography mass spectrometry (GC–MS)^68–70^ and HPLC-MS^71–74^ are commonly used for the analysis of poorly ionized lipids. Alternatively, new approaches for augmenting nonpolar lipid ionization efficiency have been developed. Direct analysis in real time (DART),^75,76^ atmospheric-pressure chemical ionization (APCI),^77^ and matrix-assisted laser desorption/ionization (MALDI)^78,79^ have been established as effective ionization methods for the analysis of nonpolar lipids. Chemical derivatization methods such as PB functionalization of C=C,^80^ carboxyl group modification with cholamine,^81^ and enzyme-assisted derivatization of hydroxyl groups^82^ can further improve ionization efficiency by introducing more readily ionizable functional groups.

These analytical challenges highlight the need for more integrated lipidomic strategies capable of delivering both structural resolution and quantitative robustness in complex biological systems. In this context, the concept of “precision lipidomics” has emerged to describe advanced MS-based workflows that enable isomer-resolved structural characterization alongside reliable and reproducible quantification. By combining selective chemical derivatization, advanced ion activation, and multidimensional separation techniques, precision lipidomics seeks to move beyond conventional lipid class-level measurements toward structurally explicit and quantitatively accurate lipid profiling.

In recent years, aziridination-enabled mass spectrometric strategies have emerged as a versatile platform to address several longstanding challenges in lipidomics, including lipid isomer characterization, quantification accuracy, and ionization efficiency. In particular, aziridination reactions selectively target C=C bonds to introduce aziridine functionalities that not only facilitate site-specific fragmentation for confident structural elucidation but also enhance ionization through the incorporation of chargeable moieties. Beyond structural characterization, integration of aziridination with complementary workflows has enabled simultaneous interrogation of C=C positions, *sn*-positional isomers, and improved quantification, highlighting its multifunctional capability (Figure 2). Notably, *N*–H aziridination provides a reactive handle for subsequent functionalization, enabling the introduction of isobaric tags for accurate relative quantification. As such, aziridination-based approaches offer a unified strategy that bridges gaps between structural resolution and quantitative analysis. This review summarizes recent advances in aziridination-enabled lipidomics, with an emphasis on *N*–H aziridination chemistry, discusses its advantages, and provides insight into the differences between the currently established methods.

**Figure 2:**
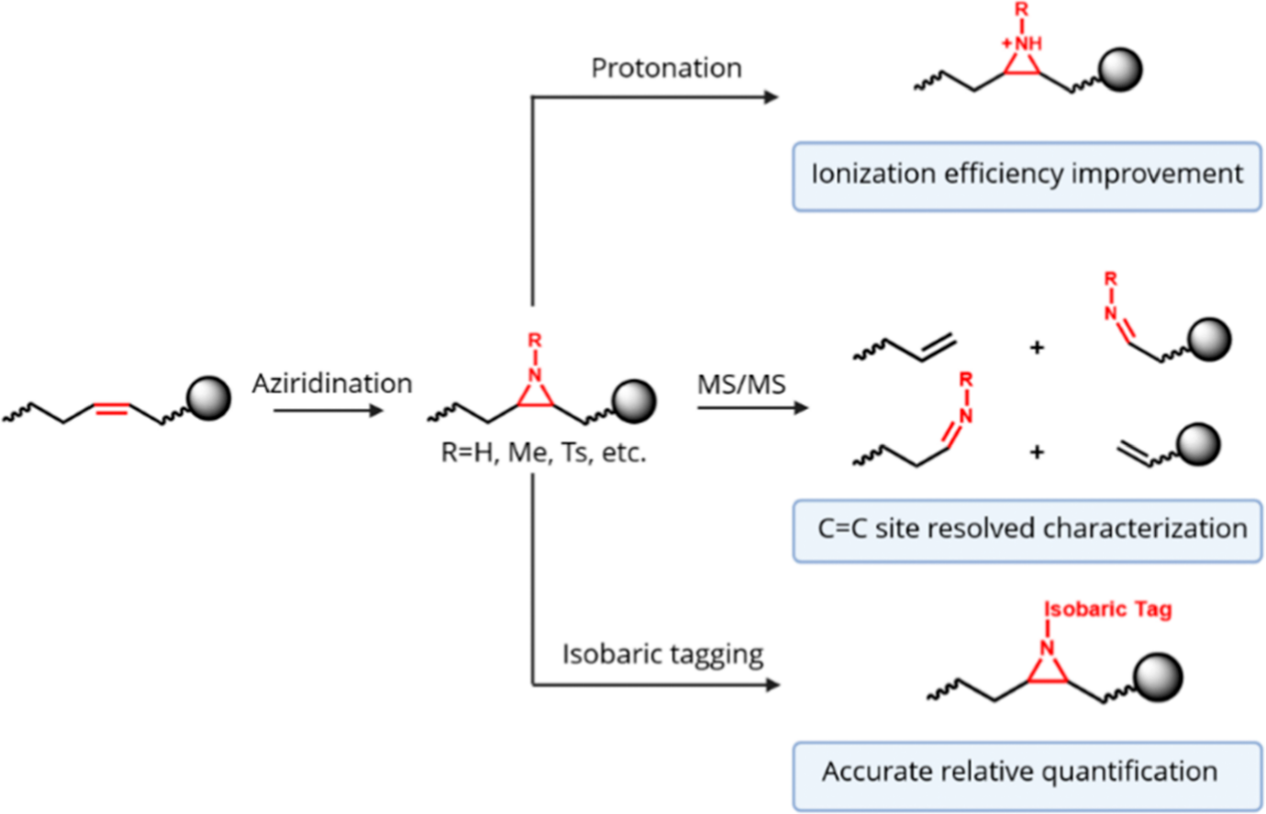
Overview of aziridination-enabled mass spectrometric strategies for lipidomics. *N*–H/*N*–R aziridination selectively targets C=C bonds to introduce aziridine groups, enhancing ionization efficiency, enabling site-specific fragmentation for structural elucidation, and serving as a handle for introducing isobaric tags for accurate relative quantification.

## Nomenclature

The lipid nomenclature in this paper follows the guidelines established by Liebisch et al.^83^ Lipid class abbreviation is followed by constituents, which are denoted by their chain length and number of C=C bonds. *Z* and *E* refer to the *cis-* and *trans-*configurations of C=C bonds, respectively. The position of C=C bonds is indicated in parentheses according to Δ-nomenclature. For *sn*-positional isomers, the separator “/” is used when the *sn*-position is known, and “_” is used when it is not. For example, PC 18:0/16:1­(6*E*) refers to a phosphatidylcholine with a saturated 18-carbon acyl chain at *sn*-1 and an unsaturated 16-carbon acyl chain at *sn*-2 with a *trans-*C=C bond between carbon 6 and 7 from the carbonyl group.

### Aziridination Chemistry and Its Translation to Lipidomics

Aziridines are important structural motifs in bioactive natural products and versatile synthetic intermediates.^84–86^ In the context of lipidomics, aziridination of C=C bonds provides a powerful ways to both enhance ionization efficiency and enable structural elucidation by introducing an aziridine ring that facilitates diagnostic fragmentation. The mechanistic pathways of aziridination reactions are reagent-dependent and have been extensively investigated in the literature.^112–118^ Early developments in aziridination chemistry largely focused on the synthesis of activated aziridines bearing electron-withdrawing protecting groups, such as tosyl and nosyl (*N*-Ts and *N*-Ns)^87–102^ groups. While these derivatives are synthetically robust, the strong *N*-sulfonyl and *N*-acyl bonds limit downstream functionalization and typically require harsh conditions for deprotection, causing challenges for modular applications.^103–108^

In contrast, unactivated aziridines bearing a free *N*–H group offer substantially greater synthetic flexibility. These species can undergo a wide range of *N*-functionalization reactions such as alkylation, acylation, sulfonylation, and carbamylation without the need for deprotection steps. This feature enables modular derivatization from a common intermediate and is particularly attractive for MS-based workflows that benefit from tunable charge introduction, isotopic labeling, or multifunctional tagging.

### Metal-Catalyzed *N*–H Aziridination of C=C Bonds

The direct synthesis of *N*–H aziridines from unactivated olefins has been enabled by Rh catalyzed nitrogen-transfer reactions using hydroxylamine-derived aminating reagents, including *O*-2,4-dinitrophenylhydroxylamine (DPH), hydroxylamine-*O*-sulfonic acid (HOSA), and *O*-(mesitylsulfonyl)­hydroxylamine (MSH) as reported by the groups of Falck, Kürti, Jat, and Tiwari.^109–111^ These methods provide efficient access to unprotected aziridines under relatively mild conditions. Subsequent efforts demonstrated that more earth-abundant metal catalysts, including Cu and Fe, can mediate analogous nitrogen-transfer reactions, improving the practicality and sustainability of these transformations.^112,113^

### Metal-Free *N*–H Aziridination

Organocatalytic *N*–H aziridination provides a metal-free approach that can improve reactivity toward unactivated (nonconjugated) C=C bonds. In previously reported metal-catalyzed *N*–H aziridination methods,^109–113^ conjugated C=C generally exhibited higher levels of reactivity than that of isolated C=C, which can limit the efficiency of these transformations for certain substrates. To address these challenges while avoiding transition metal catalysts, the Kürti group reported an organocatalytic *N*–H aziridination of unactivated olefins via transient oxaziridines generated from ethyl trifluoropyruvate using HOSA as the aminating agent.^114^ This strategy provides an effective route to *N*–H aziridines under mild, metal-free conditions with improved functional group tolerance. Complementary to organocatalytic approaches, fully catalyst-free *N*–H aziridination strategies have also been developed. The Jat and co-workers reported a metal- and additive-free protocol for the direct synthesis of *N*–H aziridines from alkenes using bench stable and hydroxylamine derived reagents as the nitrogen source.^115^ This approach eliminates the need for both transition metals and external catalysts, enhancing practicality while maintaining broad substrate compatibility.

Aziridination-based derivatization has recently been translated to lipidomics for C=C bond localization and improved detection (Table 1). Initial studies employed activated aziridine reagents. For example, the Guo group developed an *N*-tosylaziridine derivatization method for identifying C=C isomers in FAs and PCs (Figure 3a),^116^ while the Chen group reported *N*–Me aziridination strategies, including stable-isotope labeling^117^ and visible-light-driven photocatalytic systems for the derivatization of unsaturated lipids using a lab-synthesized derivatization reagent (Figure 3b).^118^

**Table 1:** Summary of N–H and *N*-Substituted (*N*–R) Aziridination Reactions^a^

aziridination reaction type	substitution	reagents	conditions	models	citation
metal-catalyzed	*N*–H	Rh_2_(esp)_2_, HOSA, pyridine	RT, 3 h	prostate cancer, colon cancer,E. coli	120, 121, 122
metal-catalyzed	*N*–Me	Rh_2_(esp)_2_, *N*-methyl-*O*-(2,4-dinitrophenyl)hydroxylamine	RT, 16–25 min	MCAO	118
organocatalytic	*N*–H	ethyl trifluoro pyruvate, HOSA, pyridine	50 °C, overnight	Alzheimer’s disease, human plasma	120
reagent-driven (iodine-mediated)	*N*-Ts	chloramine-T, sodium triiodide	RT, 15 s	Thyroid cancer, HepG2 cells, Pseudomonas putida*,*SCI	117, 125, 126
photochemical (visible light)	*N*-Ts	[(*N*-tosylimino)iodo] benzene	456 nm light, RT, 3 min	MCAO	119
catalyst-free/reagent-driven	*N*–H	TsONHBoc, NsONHHBoc, trifTsONHBoc, or Di-trifTsONHBoc	50 °C, 30 min	olive oil, CMT1A	123, 124

a Aziridination of Lipids for Mass Spectrometry.

**Figure 3:**
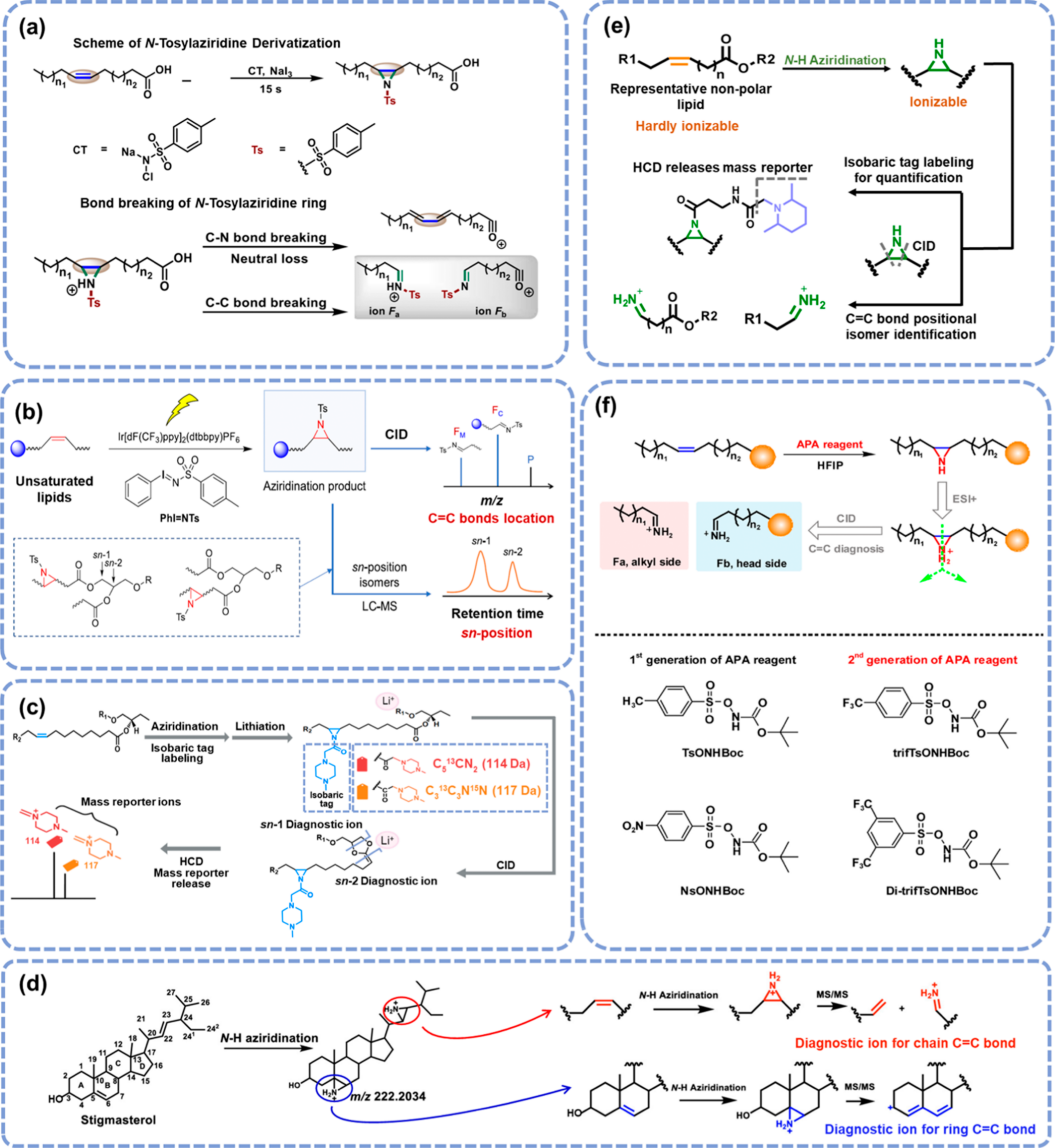
Representative aziridination-based strategies for lipid derivatization. (a) *N*-tosylaziridine derivatization of FAs enables C=C isomer identification via characteristic tandem MS fragments. (b) Visible-light photocatalytic *N*-tosylaziridination coupled with LC–MS allows for both C=C and sn-positional isomer identification. N–H aziridination of lipids enables (c) C=C isomer identification, (d) aziridine fragmentation within sterol rings via elimination of the aziridine to form a conjugated diene, leaving the sterol ring intact, and (e) *sn*-positional isomer identification with downstream functionalization for isobaric tagging and accurate relative quantification. (f) Catalyst-free APA *N*–H aziridination allows for C=C isomer identification, highlighting two generations of APA reagents. Figure 3a reproduced with permission from ref 116. Copyright American Chemical Society 2024. Figure 3b reproduced with permission from ref 118. Copyright Elsevier 2024. Figure 3c reproduced with permission from ref 121. Copyright American Chemical Society 2024. Figure 3d reproduced with permission from ref 120. Copyright American Chemical Society 2023. Figure 3e reproduced with permission from ref 120. Copyright John Wiley and Sons 2022. Figure 3f reproduced with permission from ref 123. Copyright Elsevier 2025.

The Yan group reported the first application of *N*–H aziridination to lipid analysis, enabling direct functionalization of C=C bonds in diverse lipid classes, including ST, CE, TG, FA, and PC lipids (Figure 3c,d,e).^119–121^ In contrast to *N*-sulfonylated systems, the resulting *N*–H aziridines provide a versatile handle for downstream modification, facilitating integrated workflows for structural elucidation, ionization enhancement, and quantitative analysis. Complementary strategies, such as ketone-catalyzed nitrogen transfer via oxaziridine intermediates further expand the scope of *N*–H aziridination in lipidomics.^119^ More recently, Sun lab reported aza-Prilezhaev aziridination (APA) for lipid derivatization,^122^ introducing two generations of APA reagents that significantly enhance reaction rates and conversion across diverse lipid subclasses (Figure 3f).^123^ This method is metal- and additive-free and generates predominantly monoaziridinated products, simplifying spectral interpretation. Coupled with high-resolution LC–MS and ion mobility analysis, APA enables rapid and high-sensitivity profiling of C=C positions, *sn*-isomers, and large-scale lipidome coverage; however, certain APA reagents, such as *tert*-Butyl­(((4-(trifluoromethyl)­phenyl)­sulfonyl)­oxy)­carbamate (trifTsONHBoc) and *tert*-butyl­(((4-nitrophenyl)­sulfonyl)­oxy)­carbamate (NsONHBoc) cannot be purchased commercially.

A key consideration for aziridination reactions is the degree of aziridination that a given reaction provides in polyunsaturated lipids; for example, a reaction that yields only a singly aziridinated product will produce a higher signal and will be simpler to analyze than one that produces products with different levels of aziridination. The more products generated, the more complicated the analysis will be. Notably, rhodium-catalyzed aziridination has been reported to favorably produce multiply aziridinated products. When lipids have multiple C=C on the same chain, the monoaziridination product has been found to be consistently favored for the *N*–Me aziridination, while the diaziridinated product is favored when C=C are located on different aliphatic chains;^117^ however, in the case of rhodium-catalyzed *N*–H aziridination, the diaziridination product was dominant for the *N*–H aziridination of PC 16:0/20:4.^121^ For aziridination with chloramine-T,^116^ ethyl trifluoropyruvate,^119^ APA,^122^ and photocatalysis,^118^ the monoderivatization product of polyunsaturated lipids was dominant.

Conversion rates vary by approach. Derivatization with chloramine-T^117^ and APA^122,123^ were reported to yield nearly full conversion of reactants. Optimized rhodium-catalyzed *N*–H experiments yielded 90% conversion.^121^ Other reactions have reported more limited conversion; for aziridination using ethyl trifluoropyruvate, representative TG, CE, and FA lipids had conversion percentages of 50–88.2%, but PC 16:0/18:1 (9) only had a conversion of 6%,^119^ while photocatalyzed aziridination was calculated to be ∼20% after 3 min and ∼35% after 25 min^118^

### Structural Characterization of Lipid Isomers

#### C=C Bond Positional Isomers

Aziridination-based strategies enable consistent localization of C=C bonds in unsaturated lipids. Regardless of whether the aziridine is N-substituted or *N*–H, these methods share a common fragmentation pathway under CID. Upon activation, the aziridine ring undergoes ring opening followed by cleavage along the original C=C bond to generate a pair of diagnostic imine fragments that encode the C=C position (Figure 2).^116–125^ In practice, however, both diagnostic ions are not always observed. For certain C=C positions, the fragment corresponding to the terminal end of the acyl chain may fall below the low-mass cutoff of ion trap mass analyzers, leading to incomplete detection.^119^

Distinct fragmentation behavior of aziridines is observed when the C=C bond resides within a sterol ring rather than an aliphatic chain (Figure 3f). In these cases, CID-induced aziridine ring opening does not break the sterol ring; rather, the aziridine moiety is eliminated to produce a conjugated diene.^120^ This divergence in fragmentation pathways provides a useful basis for distinguishing between sterol isomers. For example, 7-dehydrocholesterol and desmosterol can be differentiated based on their characteristic fragmentation patterns, reflecting the presence of two C=C bonds within the sterol B-ring in the former and a combination of ring and side-chain unsaturation in the latter.^120^

### *sn*-Positional Isomers

Unlike C=C bond positional isomers, *sn*-positional isomers cannot be universally resolved by the aziridination approach alone and typically require integration with complementary separation or activation strategies. Methods for *sn*-isomer identification rely on the generation of diagnostic ions corresponding to the neutral loss of ketene or free fatty acid from the glycerophospholipid backbone under CID.

Analysis of *sn*-positional isomers can be enabled by integrating aziridination-assisted mass tagging with lithiation (Figure 3d).^121^ This strategy does not require an orthogonal technique to separate *sn*-isomers prior to analysis and can be used with a shotgun approach or HPLC-MS. Lipids are functionalized with an *N*–H aziridine by Rh-catalyzed aziridination, which is then labeled with an amine-reactive isobaric tag for relative and absolute quantification (iTRAQ). Lithium is introduced to the spray solution in one of two ways: for direct infusion, lithium acetate is introduced directly into the sample, while for HPLC-MS, lithium acetate is introduced into the postcolumn flow path via a tee union. Lithium-adducted ions were found to exhibit an enhanced fragmentation efficiency in subsequent tandem analyses. During analysis, MS^2^ fragments off the headgroup to form a dioxolane, which then undergoes a dioxolane-induced cross-ring cleavage in MS^3^ to form position-specific fragments. These diagnostic ions can fragment in MS^4^ to produce isobaric reporter ions that can be used for quantification.^121^

*N*-Tosylaziridination by photocatalysis has been combined with LC–MS to provide *sn-*isomer resolution (Figure 3b).^118^ The introduction of the bulky tosylaziridine to one of the fatty acyl chains enhances chromatographic separation, allowing *sn*-positional isomers to elute as distinct peaks at unique retention times in LC. The two eluent peaks can be identified by examining the ratios between the ions corresponding to the neutral loss of ketene and that of free fatty acid. A higher abundance of ketene ions than free fatty acid ions corresponding to the loss of the same chain indicates an *sn*-1 position, while the inverse indicates an *sn*-2 position.^118^

More recently, APA has been coupled with ion mobility spectrometry using a U-shaped mobility analyzer (UMA) to further enhance *sn*-isomer differentiation.^122^ In this workflow, lipids derivatized by APA are separated based on subtle structural and stereochemical differences, with sodiated species exhibiting improved mobility resolution. Although initial tandem MS (MS^2^) produces *sn*-diagnostic ions with limited efficiency due to competing headgroup loss, this limitation is mitigated through a “pseudo MS”^3^ strategy inspired by previous work.^25,29^ Here, headgroup cleavage occurs in the segmented multipole guide, prior to mass selection, enabling subsequent fragmentation of backbone-specific ions to generate structurally informative fragments with enhanced intensity. GPLs with different head groups could still be roughly distinguished with this method because of UMA separation. It was noted that the separation between lipid isomers could be further augmented through either derivatizing an *N*–R aziridine or using a two-step process to achieve *N*–R aziridines from *N*–H; however, the separation provided by *N*–H aziridination alone was sufficient for identifying *sn*-lipid isomers.^122^

### *Cis–Trans* Configuration Isomers

Although aziridination alone is not sufficient for distinguishing between *cis*- and *trans*-isomers, stereospecific aziridination via chloramine-T can be used to introduce a tosylaziridine group that, when used in conjunction with tandem ion mobility-mass spectrometry (IM-MS), can exaggerate structural differences between the two isomers to allow for *cis–trans* identification.^125^ It was proposed that the spatially selective *p*-toluenesulfonyl group causes the formation of diastereoisomers via intramolecular hydrogen bonding, and steric hindrance influences the ratios of isomers formed. Following IM-MS, *cis*-C=C bonds show a single peak in the extracted ion mobilogram (EIM), whereas *trans*-C=C bonds show two peaks. The method was established to function for both mono- and polyunsaturated FAs, FA methyl esters, FA glycerides, and higher fatty alcohols.^125^

### Quantitative Lipidomics Enabled by Aziridination

While many aziridination-based workflows have demonstrated semiquantitative analysis of lipids,^118,120,122,123^ achieving accurate quantification remains challenging. Traditional approaches rely on stable-isotope-labeled internal standards; however, their application to aziridinated lipids is often limited by the availability of structurally matched standards.^116^ More critically, for unknown or uncharacterized lipid species, the preparation of corresponding standards is often impractical or infeasible, further hindering accurate quantification. One strategy to overcome the need for reference standards is derivatizing lipids with isobaric tags, labeling reagents originally established in quantitative proteomics.^126^ Tagged samples can be multiplexed so that each analyte generates a single peak in MS^1^ but generates unique mass reporter ions at different *m*/*z*’s, the intensities of which can be used for accurate relative quantification.^119,121,124^ This method of combined sample analysis negates the inaccuracy caused by matrix effects, enhances throughput, and reduces technical variance.^127,128^

An early implementation of this concept leveraged *N*–H aziridination of various lipid classes for isobaric tagging (Figure 4a).^119^ In this approach, unsaturated lipids are first functionalized via Rh-catalyzed *N*–H aziridination, generating a reactive amine handle for subsequent conjugation with amine-active isobaric mass tags. After sample pooling, HPLC-MS analysis enables simultaneous acquisition of structural and quantitative information: CID produces diagnostic fragments for C=C localization, while higher-energy collisional dissociation releases reporter ions for quantification.^119^ This approach broadens lipid class coverage and highlights the modularity of *N*–H-aziridination.

**Figure 4:**
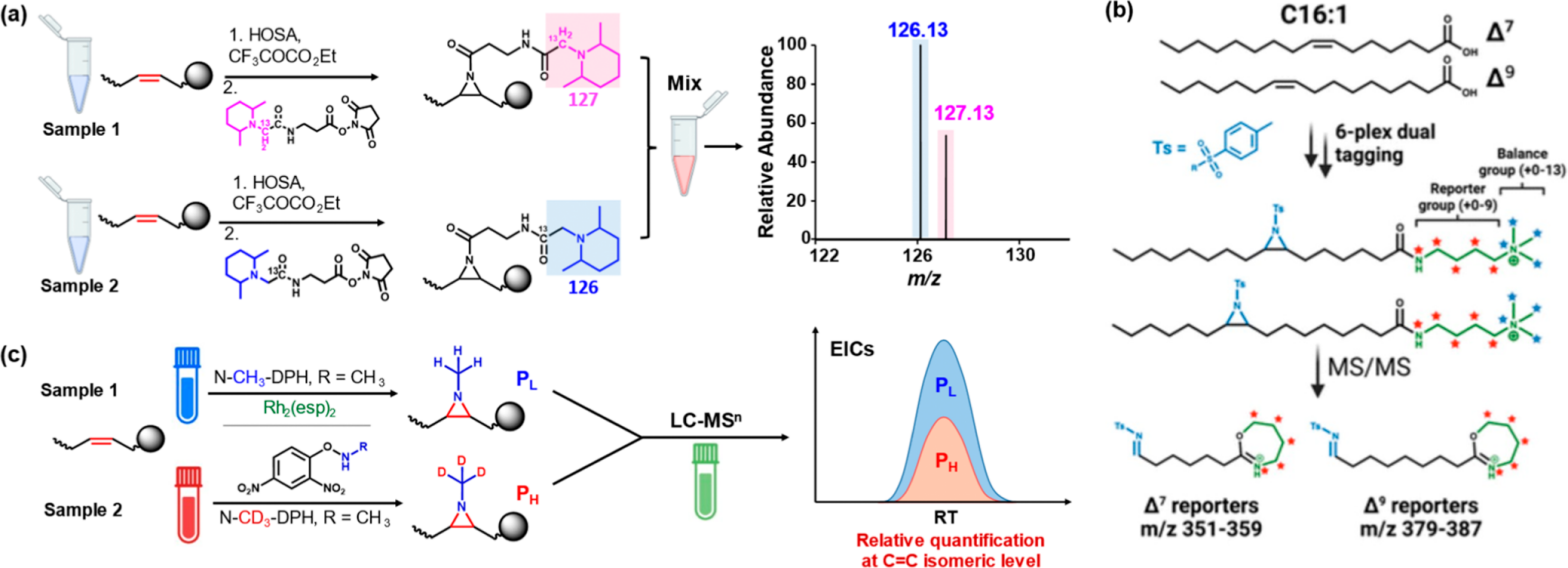
Generalized workflows of aziridination-assisted accurate relative quantification methods. (a) *N*–H aziridination is used sequentially with IMT labeling of two separate samples, which are then combined for a single analysis. Under tandem MS, reporter ions at *m*/*z* 126.13 and 127.13 are released and their ratios are used for quantification. (b) The headgroups of FAs are labeled with 6-plex dual tags while their C=C bonds are aziridinated with an *N*-Ts group. The tags can fragment to generate reporter ions for quantification, while the *N*-Ts aziridine enables C=C bond identification. (c) Two samples are separately labeled with either unlabeled or deuterated *N*–Me aziridines and then pooled for LC–MS analysis. The area under the two chromatographic curves can be used for quantification. Figure 4a reproduced with permission from ref 119. Copyright John Wiley and Sons 2022. Figure 4b reproduced with permission from ref 124. Copyright Royal Society of Chemistry 2023. Figure 3c reproduced with permission from ref 117. Copyright American Chemical Society 2024.

This method was subsequently extended to the quantification of *sn*-positional isomers through integration with iTRAQ labeling and lithiation.^121^ Following sequential aziridination and tagging, lipids are analyzed under lithiated conditions. In this workflow, MS^2^ induces headgroup loss, MS^3^ generates *sn*-position-specific fragments via dioxolane-mediated cleavage, and MS^4^ releases reporter ions for quantification. This multistage strategy enables simultaneous determination of C=C location, *sn*-position, and their relative abundances, although sensitivity limitations may arise in shotgun workflows due to reduced reporter ion intensity at higher MS^n^ levels.^121^

Isobaric tagging for accurate relative quantification via a carboxylic acid-active tag was achieved alongside tosylaziridination for C=C isomer resolution for the study of free fatty acids (FFAs) (Figure 4b).^124^ In this approach, FFAs are first labeled with 6-plex isobaric tags, and then all samples are combined for tosylaziridination of the C=Cs before LC–MS analysis. In MS,^2^ the aziridine fragments to form the diagnostic imine ion while the mass balancer of the isobaric tag simultaneously cleaves off, leaving behind the mass reporter as a cyclization product. Because the diagnostic fragment and mass reporter are generated as a single ion, C=C bond isomers can be accurately quantified.

In parallel, stable isotope labeling approaches^129^ have also been adapted for aziridination-enabled lipid quantification (Figure 4c). A representative approach employs a stable-isotope *N*–Me aziridination reaction that enables simultaneous structural characterization and relative quantification of unsaturated lipid isomers.^117^ In this method, a 1-methylaziridine moiety is introduced in a single step, serving a dual function as both a reactive handle for C=C bond localization and an isotopic label for quantification. Upon tandem mass spectrometric analysis, diagnostic fragmentation enables identification of C=C positions, while isotopic differences between labeled species allow accurate relative quantification based on extracted ion chromatogram peak areas. This bifunctional labeling strategy has been demonstrated for large-scale analysis of unsaturated lipids across multiple classes, including FAs, phospholipids, glycerides, and CEs, enabling isomer-resolved quantification in complex biological samples.^117^

## Enhancement of Ionization Efficiency via Aziridination

Aziridination has consistently been shown to enhance the ionization efficiency of lipids across a range of methodologies.^116,117,119,120,122,123^ Nonpolar lipids ionize poorly by ESI and can be challenging to detect on their own. Aziridination introduces a protophilic group to lipids, which can significantly improve positive-mode detection of lipids that were otherwise low-abundance or undetectable by MS in positive mode (Figure 5). Both *N*–H^119,120,122,123^ and substituted^116,117^ aziridines have been observed to provide this benefit. Notable improvement in positive-mode ionization efficiency has been observed in aziridinated ST, CE, TG, FA, and DG lipids.^117,119,120^ Even for lipids that can be detected by MS without modification, aziridination lowered the limit of detection relative to the unmodified lipid, aiding in the analysis of low-abundance poorly ionized lipids.^117,119^

**Figure 5:**
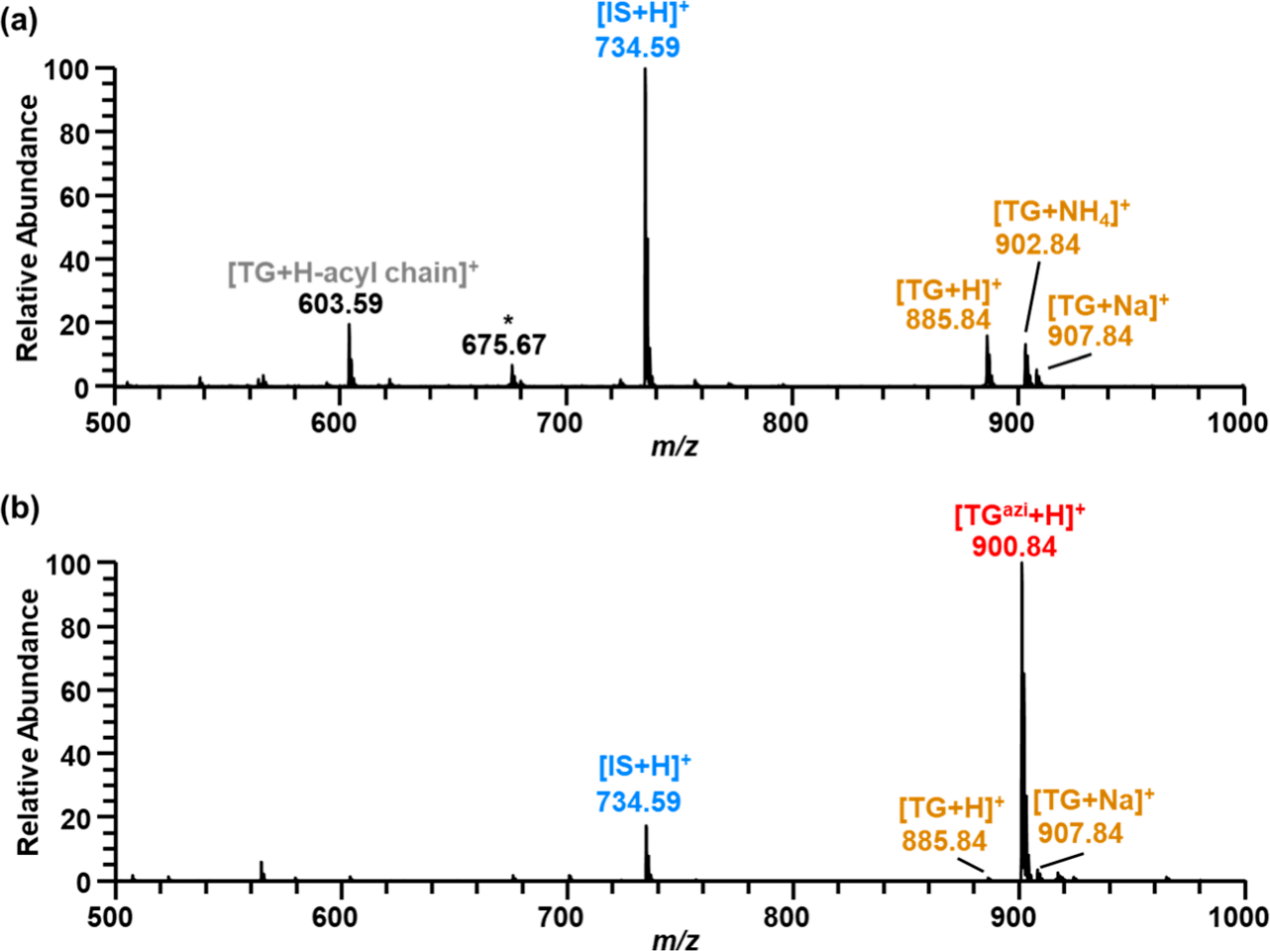
Aziridination of nonpolar lipids significantly improves the ionization efficiency in MS analysis. Full MS of TG 18:1(9)/18:1(9)/18:1(9) (a) before and (b) after aziridination. Internal standard PC 16:0/16:0 (*m*/*z* 734.59) at 10 μM was used. * denotes a background peak from the solvent system. Figure adapted with permission from ref 119. Copyright John Wiley and Sons 2022.

### Applications of Aziridination in Structural and Quantitative Lipidomics

Aziridination has been leveraged to study lipid isomers in many applications. Hirtzel et al. used *N*–H aziridination with HPLC-MS to analyze changes in nonpolar lipids in prostate cancer. It was found that CEs and STs were upregulated up to 4.49-fold in the cancerous samples, while TGs were downregulated as much as 6.06-fold, alongside identification of C=C positions.^120^ Aziridination was used with LC–MS by Li et al. to generate a database of *m*/*z*, retention time, and MS^2^ for 393 unsaturated lipids in Charcot-Marie-Tooth disease type 1A (CMT1A).^123^ After comparison to the control model, 40 lipids were identified as potential diagnostic markers for CMT1A. A semiquantitative analysis of the relative intensity of C=C diagnostic ions determined that the ratio of n-7/n-9 C=C isomers was upregulated in CMT1A, while the ratio of n-3/n-6 was downregulated (Figure 6).^123^ Work from Zhang et al. studied changes in PCs, lysophosphatidylcholines (LPCs), and FAs in tumor and paratumor thyroid tissues with C=C isomer resolution using tosylaziridination and direct infusion.^116^ In general, FAs, LPCs, and PCs were found to be more abundant in the tumor sample than that of the paratumor, and in C=C isomer analysis, some species of n-6/n-3 FAs were found to be closely related to thyroid cancer (Figure 7). Using reference standards, 12 species of unsaturated FAs were accurately quantified.^116^

**Figure 6:**
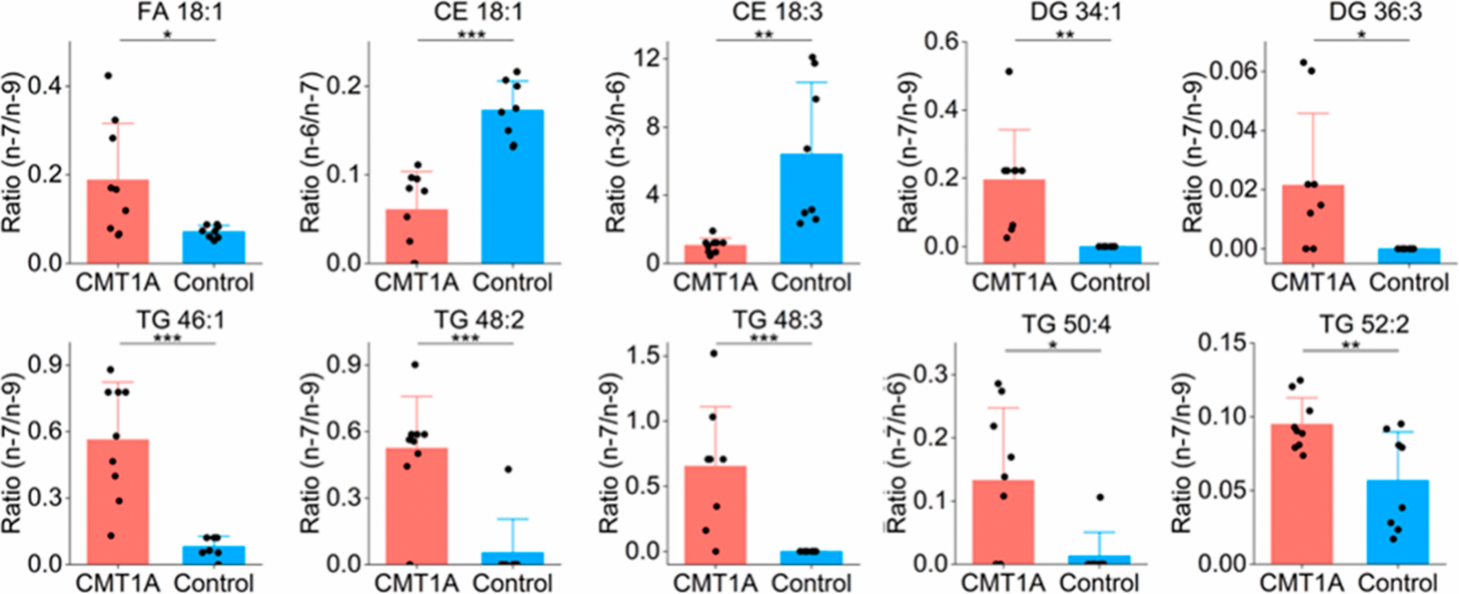
Comparison of C=C isomer ratios of unsaturated lipids in CMT1A and control patient samples. *p < 0.05, **p < 0.01, ***p < 0.001. Reproduced with permission from ref 123. Copyright Elsevier 2025.

**Figure 7:**
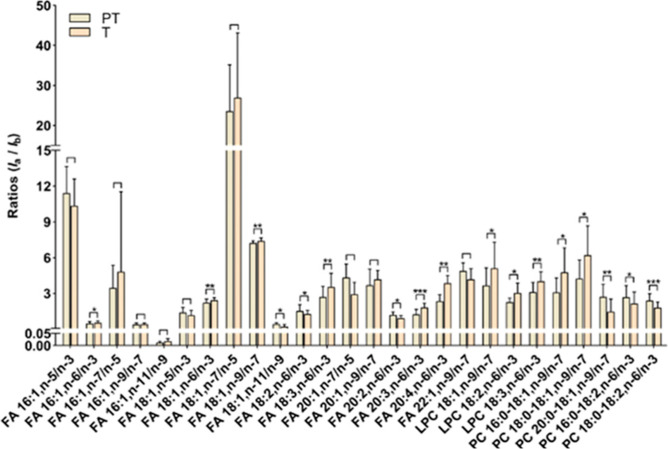
Analysis of the relative isomer ratio between different families of FAs between tumor and paratumor thyroid tissues. Reproduced with permission from ref 116. Copyright American Chemical Society 2022.

Wan et al. demonstrated the *sn*-isomer-resolving ability of aziridination-assisted LC–MS in a middle cerebral artery occlusion (MCAO) model of focal ischemia.^118^ It was found that the ratio of PC 16:0/18:1(9) to PC 18:1(9)/16:0 was significantly higher in ischemic brain tissue than the control model, and isomers with C=C at the *sn*-2 position were generally more abundant than those with C=C on the *sn*-1 chain, suggesting that the ratios of *sn*-isomers in cerebral ischemia may change due to dysregulated biosynthesis or metabolism.^118^ In another report, Li et al. used aziridination with UMA-QTOF MS to analyze low-polarity lipids (12 FAs, 14 DGs, 39 TGs, and 5 SEs) in olive oil with C=C and *sn*-position resolution.^122^ Tang et al. leveraged asymmetric aziridination with IM-MS to study *cis–trans* configuration changes in the lipidome of *Pseudomonas putida*, a solvent-resistant bacterium, following the activation of *cis–trans* isomerase (Cti) to promote isomerization.^125^ It was found that when 1-octanol was introduced to activate Cti, the ratio of FA 18:1 (9) to FA 18:1 (11) did not change significantly; however, the ratio of FA 18:1 (9*E*) to FA 18:1 (9*Z*) significantly increased after the stimulation of Cti with 0.4% 1-octanol. The same method was used to study changes in the lipidome along the spinal cord in rats with spinal cord injury (SCI). A notable change in C=C bond positional isomer ratios was seen 1 day after SCI when compared to healthy rats, with changes in C=C bonds occurring mostly around the site of injury. Three days after injury, the isomer changes reverted to the normal levels. The FAs studied were found to maintain their *cis* configuration in SCI.^125^

Work by Armbruster et al. combined accurate relative quantification via carboxylate tags with C=C identification via aziridination to study FFA dysregulation in HepG2 cells following treatment with CAY10566, a stearoyl-CoA desaturase (SCD1).^124^ Treated cells contained lower levels of monounsaturated FAs due to SCD1’s role in converting fatty acids to their Δ9 counterparts, while saturated fatty acids were largely unaffected.^124^ Yang et al. used *N–*H aziridination to facilitate amine-active isobaric tag labeling of CEs for accurate relative quantification and characterization of lipids in human plasma and Alzheimer’s disease (AD) mouse serum.^119^ In the human serum analysis, lipid extracts were labeled separately and prepared in two solutions at a fixed concentration ratio of 2:1 to validate the method on a biological sample. Following the successful demonstration of lipid quantification in the expected ratio, the method was applied to AD mouse and control mouse serum. A total of 17 CE isomers in AD were quantified relative to the control, with 6 CEs showing concentration changes by *a* factor of 2 of more.^119^ Later work by Yang et al. used aziridination-assisted iTRAQ labeling to accurately quantify *sn*-isomers of GPLs in *E. coli* and colon cancer human plasma.^121^ *E. coli* samples were prepared in predetermined concentration ratios as a proof of method, then colon cancer human plasma was quantified against healthy human plasma. A total of 17 *sn*-positional isomers were characterized and quantified in the serum samples; 7 PC isomers significantly changed in abundance—by at least 2-fold—in colon cancer plasma relative to healthy plasma (Figure 8).^121^

**Figure 8:**
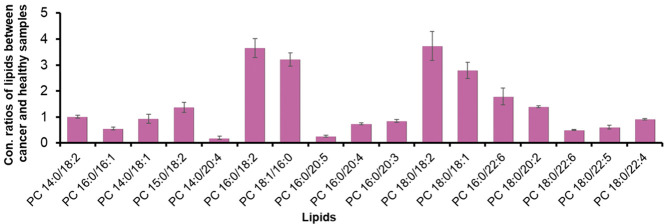
Concentration ratios of PC lipids between colon cancer and healthy samples after identifying *sn*-positional isomers. Reproduced with permission from ref 121. Copyright American Chemical Society 2024.

Feng et al. analyzed unsaturated lipids with stable-isotope *N*–Me aziridination in human serum and a focal ischemia model of MCAO in mice.^117^ For human serum, a workflow was developed in which underivatized lipids were analyzed by LC–MS/MS to generate a putative list, which informed a targeted lipidomic analysis using both stable-isotope and nonisotopic *N*–Me aziridination. Using this method, 551 serum lipids were identified, with 468 lipids identified at the C=C level. The accuracy of the method’s relative quantification was evaluated using equal amounts of mouse brain tissue derivatized with the light and heavy aziridination. After validation, normal and ischemic brain tissues were relatively quantified. Significant differences in several unsaturated lipids were identified between the two tissues, including an increase of certain phospholipids and a decrease in some triglycerides. Certain C=C positional isomers differed significantly between the normal and ischemic tissues, while the abundance of lipids with the same chain length but different C=C position remained unperturbed (Figure 9).^117^

**Figure 9:**
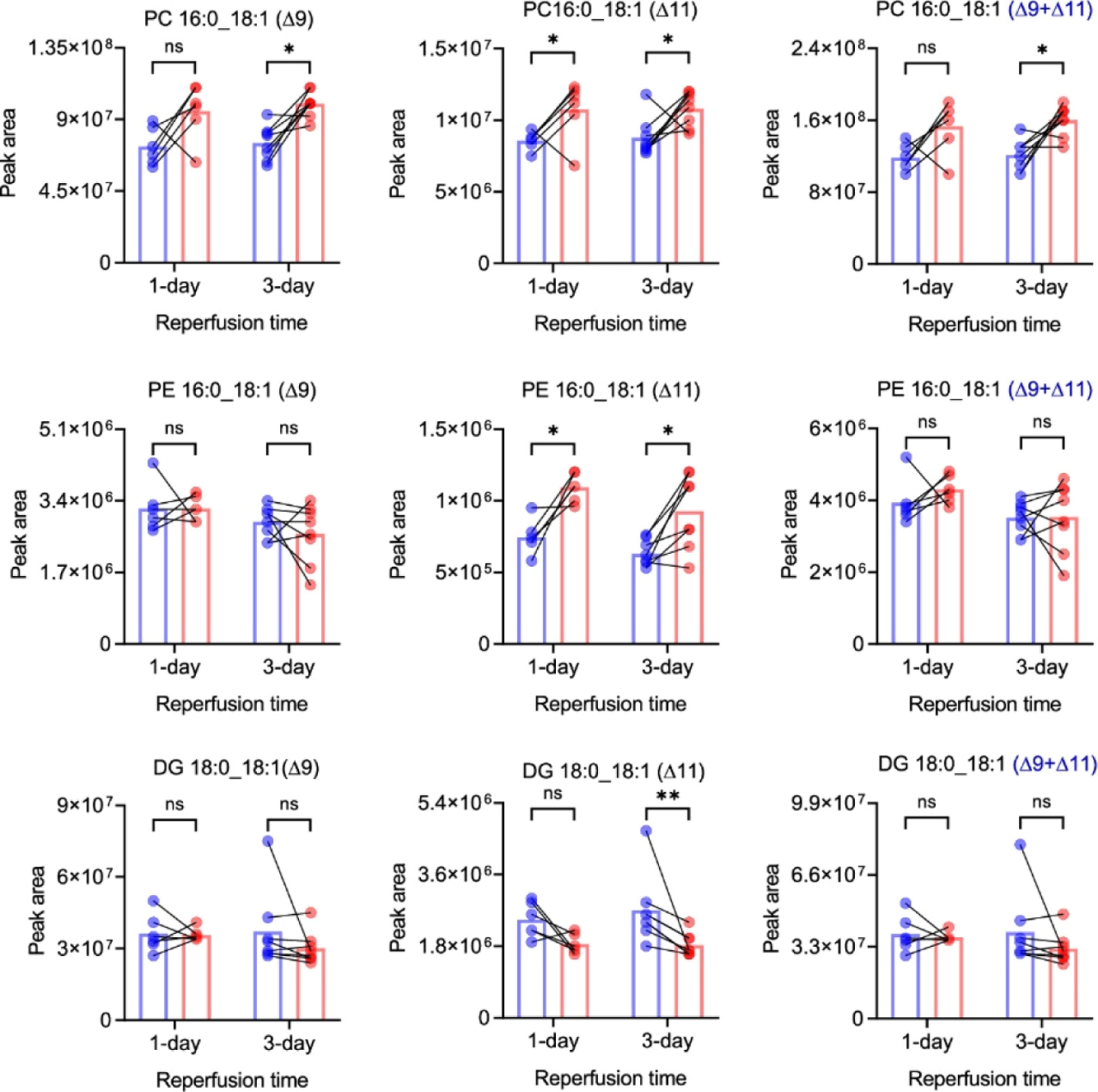
Relative quantification of lipid isomers in MCAO mice model brain tissues. Reproduced with permission from ref 117. Copyright American Chemical Society 2024.

#### Reaction-Based Derivatization in Lipidomics

Reaction-based derivatization methods have experienced great interest in recent years as they do not necessitate the use of specialized instrumentation. Many olefin-targeting reactions have been leveraged to overcome the lipidomic challenges. These include reaction-based derivatization approaches such as epoxidation, PB photocycloaddition, ozonolysis, and other chemoselective transformations, as we mentioned in the Introduction. Each methodology offers distinct advantages with respect to structural information, instrumental requirements, reaction scope, analytical throughput, and sensitivity. A comprehensive comparison of all available approaches is beyond the scope of this Review; however, it is useful to place aziridination in the context of other reaction-based derivatization strategies that similarly rely on chemical modification of C=C bonds prior to MS analysis.

Epoxidation is one of the most widespread methods because of its operational simplicity and broad applicability. Unsaturated lipids can be epoxidized offline using *meta-*chloroperoxybenzoic acid (*m*-CPBA)^44^ or online via low-temperature plasma (LTP)^41,42,130^ or electro-epoxidation during nESI.^45^ Electro-epoxidation can be switched on and off by adjusting ESI voltage using a large-orifice emitter, allowing for the collection of both derivatized and underivatized spectra in a single run,^45^ an important advantage over bulk derivatization approaches. PB photocycloaddition has also been extensively explored for C=C bond localization and can be implemented in online workflows.^47,131^ Depending on the reagent employed, PB derivatization can additionally improve ionization efficiency^132^ and has been demonstrated to facilitate the identification of *sn*-positional isomers.^25,133^

Aziridination shares several advantages with these established derivatization methods while offering unique capabilities. Like epoxidation and PB derivatization, aziridination generates diagnostic fragmentation patterns that enable C=C bond localization. However, the introduction of a nitrogen-containing aziridine ring can substantially enhance the ionization efficiency of nonpolar lipid classes, a feature that is generally not observed with epoxidation. Furthermore, N–H aziridination introduces a synthetically versatile amine functionality that can be readily exploited for subsequent chemical modification.^119,121^ This feature enables the incorporation of reporter tags and other functionalities, expanding opportunities for quantitative analysis and targeted lipid characterization. As demonstrated in recent studies,^121^ aziridination can also facilitate the differentiation of *sn*-positional isomers and support quantitative workflows. These characteristics highlight the complementary role of aziridination within the broader toolbox of lipid double-bond derivatization strategies and help explain the growing interest in its application to lipidomics.

## Conclusion

Mass spectrometry has become an indispensable tool for lipidomics; however, conventional MS alone remains insufficient for comprehensive structural characterization and accurate quantification of lipid isomers. Aziridination-assisted mass spectrometry is a young but promising approach to lipidomics. A diverse array of reactions have been established, each with its own advantages and considerations, including reaction time, yield, temperature, and degree of derivatization. All established strategies provide C=C resolution and have the potential to improve ionization efficiency in the positive mode, which is a boon to methods that seek to leverage aziridination for an additional purpose, such as *sn*-isomer identification or accurate quantification without reference standards. Eliminating the necessity for reference standards in quantification is particularly appealing, as many complex lipids or unknown lipids do not have adequate synthetic standards commercially available.^3^ *N*–R aziridination can be used to directly insert moieties with strategic analytical functions, while *N*–H aziridination provides a functional group to be targeted for further derivatization, which can expand methods previously reserved for specific analytes to a broad range of unsaturated lipids. Aziridination-assisted mass spectrometry has been demonstrated on a variety of applications and presents a new avenue for lipidomic discovery.
